# Cost-Utility Analysis of Direct-Acting Antivirals for Treatment of Chronic Hepatitis C Genotype 1 and 6 in Vietnam

**DOI:** 10.1016/j.jval.2020.03.018

**Published:** 2020-09

**Authors:** Ong The Due, Ammarin Thakkinstian, Montarat Thavorncharoensap, Abhasnee Sobhonslidsuk, Olivia Wu, Nguyen Khanh Phuong, Usa Chaikledkaew

**Affiliations:** 1 Mahidol University Health Technology Assessment (MUHTA) Graduate Program, Mahidol University, Bangkok, Thailand; 2 Health Strategy and Policy Institute, Ministry of Health, Hanoi, Vietnam; 3 Department of Clinical Epidemiology and Biostatistics, Faculty of Medicine Ramathibodi Hospital, Mahidol University, Bangkok, Thailand; 4 Social and Administrative Pharmacy Division, Department of Pharmacy, Faculty of Pharmacy, Mahidol University, Bangkok, Thailand; 5 Division of Gastroenterology and Hepatology, Department of Medicine, Faculty of Medicine Ramathibodi Hospital, Mahidol University, Bangkok, Thailand; 6 Health Economics and Health Technology Assessment (HEHTA), Institute of Health and Wellbeing, University of Glasgow, Glasgow, United Kingdom

**Keywords:** chronic hepatitis C, direct-acting antivirals, economic evaluation, genotype 6, Vietnam

## Abstract

**Objective:**

Very few cost-utility analyses have either evaluated direct-acting antivirals (DAAs) on hepatitis C virus (HCV) genotype 6 patients or undertaken societal perspective. Recently, DAAs have been introduced into the Vietnamese health insurance drug list for chronic hepatitis C (CHC) treatment without empirical cost-effectiveness evidence. This study was conducted to generate these data on DAAs among CHC patients with genotypes 1 and 6 in Vietnam.

**Methods:**

A hybrid decision-tree and Markov model was employed to compare costs and quality-adjusted life-years (QALYs) of available DAAs, including (1) sofosbuvir/ledipasvir, (2) sofosbuvir/velpatasvir, and (3) sofosbuvir plus daclatasvir, with pegylated-interferon plus ribavirin (PR). Primary data collection was conducted in Vietnam to identify costs and utility values. Incremental cost-effectiveness ratios were estimated from societal and payer perspectives. Uncertainty and scenario analyses and value of information analyses were performed.

**Results:**

All DAAs were cost-saving as compared with PR in CHC patients with genotypes 1 and 6 in Vietnam, and sofosbuvir/velpatasvir was the most cost-saving regimen, from both societal and payer perspectives. From the societal perspective, DAAs were associated with the increment of quality-adjusted life-years by 1.33 to 1.35 and decrement of costs by $6519 to $7246. Uncertainty and scenario analyses confirmed the robustness of base-case results, whereas the value of information analyses suggested the need for further research on relative treatment efficacies among DAA regimens.

**Conclusions:**

Allocating resources for DAA treatment for HCV genotype 1 and 6 is surely a rewarding public health investment in Vietnam. It is recommended that the government rapidly scale up treatment and enable financial accessibility for HCV patients.

## Highlights

• There have already been several cost-utility analyses of direct-acting antiviral (DAA) regimens conducted, but very few have either evaluated DAA regimens on hepatitis C virus (HCV) genotype 6 patients or undertaken a societal perspective. Our study has addressed this gap in knowledge by being among the first cost-utility analyses of DAA regimens on HCV genotype 6 patients using a societal perspective.
• In this study, sofosbuvir/velpatasvir (SOF/VEL), sofosbuvir/ledipasvir (SOF/LDV), and sofosbuvir plus daclatasvir (SOF+DCV) are found to be cost-saving compared with pegylated-interferon plus ribavirin (PR) in HCV patients with genotypes 1 and 6 in Vietnam, from both societal and payer perspectives. Among 3 DAAs, SOF/VEL is the most cost-effective, followed by SOF/LDV as the second, and SOF+DCV as the third. These results remain robust across all government’s copayment rates for DAAs. Our result would be particularly useful for countries with a high prevalence of HCV genotype 6 such as Laos, Cambodia, Myanmar, or Thailand.

## Introduction

In Vietnam, an estimated 1%-2% of the total population is infected with hepatitis C virus (HCV)^1^, ^2^, ^3^, ^4^, ^5^ and there are 6 new HCV infections per 100 000 persons annually.^6^ In addition, Vietnam is also found to have the unique HCV genotype 6 as the most prevalent genotype, followed by HCV genotype 1, which together account for more than 85% of HCV infections.^7^, ^8^, ^9^, ^10^, ^11^ It should be noted that genotype 6, which is mainly found in Southeast Asia, and only accounts for 2% of HCV infections in the world, is relatively restricted in geographical extent.^12^^,^^13^

Since 2019, the Ministry of Health has implemented a new health insurance drug list, where direct-acting antiviral (DAA) regimens have been introduced for the first time. Three regimens were included: (1) sofosbuvir/ledipasvir (SOF/LDV), (2) sofosbuvir/velpatasvir (SOF/VEL), and (3) sofosbuvir plus daclatasvir (SOF+DCV), while also retaining the old standard of HCV treatment, pegylated-interferon plus ribavirin (PR).^14^ Nevertheless, there has been no empirical evidence on the cost-effectiveness of the newly included DAA regimens in the Vietnamese population. Although there have already been several cost-utility analyses (CUAs) of DAA regimens conducted, very few have evaluated DAA regimens on HCV genotype 6, been conducted in low- and middle-income countries (LMICs), or undertook a societal perspective, according to the results of recently published systematic reviews on CUAs of DAA regimens.^15^, ^16^, ^17^, ^18^ This has posed a significant gap in knowledge on the adaptability of the new DAAs. Therefore, this CUA was conducted on an HCV population genotype 1 and 6 in Vietnam, from both societal and payer perspectives. The cost-effective evidence generated by this study may inform policy makers in revising the drug list in the coming years and revising the HCV treatment guidelines. Moreover, this study may contribute to the global knowledge on the cost-effectiveness of DAA regimens, as being among the first CUAs of DAA regimens that considered HCV genotype 6 patients in an LMIC using a societal perspective.

## Methods

### Target Population

The model simulated cohorts of patients infected with HCV genotype 1 and 6 in Vietnam because these 2 genotypes are the most prevalent.^7^, ^8^, ^9^, ^10^, ^11^ The mean age of the HCV patients was assumed to be 50 years old, based on the results from recent surveys in Vietnam.^9^, ^10^, ^11^^,^^19^^,^^20^ The patients had no comorbidity and were at either non-cirrhotic chronic hepatitis C (CHC) or compensated cirrhosis (CC) health states—the 2 heath states that are eligible for HCV antiviral treatment in Vietnam.^21^^,^^22^

### Model Structure

A hybrid of decision-tree and Markov models adapted from Kapol et al^23^ was applied, which was validated by clinical experts in Vietnam’s National Hospital of Tropical Diseases.

**Figure 1:**
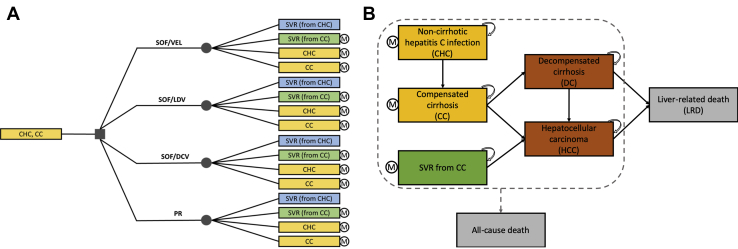
The hybrid decision-tree and discrete-time, state-transition Markov model.

Patients entered a decision tree at the initiation of treatment and were chosen to receive either DAA regimens or PR. Upon completion of each regimen, patients could move to 1 of corresponding 4 health states based on their status of sustained virologic response (SVR)—the indication of successful treatment. CHC and CC patients who achieved SVR were assumed to be cured, but CC patients could still progress to hepatocellular carcinoma (HCC),^24^, ^25^, ^26^, ^27^ although at a slower rate, and patients who failed to achieve SVR would continue to progress over time. Therefore, all patients, except those who achieved SVR from CHC, moved to a discrete-time state-transition Markov model for natural disease progression (Fig. 1).

CC indicates compensated cirrhosis; CHC, non-cirrhotic chronic hepatitis C; DC, decompensated cirrhosis; DCV, daclatasvir; HCC, hepatocellular carcinoma; LDV, ledipasvir; LRD, liver-related death; PR, pegylated-interferon plus ribavirin; SOF, sofosbuvir; SVR, sustained virologic response; RBV, ribavirin; VEL, velpatasvir.

The Markov model was based on the natural history of hepatitis C, and the classification of health states was in line with the current treatment guidelines in Vietnam.^22^ There were 6 mutually exclusive health states, including an SVR state achieved after successful treatment of CC, 4 disease states (ie, CHC, CC, decompensated cirrhosis [DC], and HCC), and a dead state, which was liver-related death (LRD). The age-specific probabilities of all-cause mortality were applied to all health states, whereas only patients with DC and HCC would die from liver-related mortality. The model simulation ended when all patients in the cohort died (ie, reached 100 years old according to the latest life table of Vietnam).^28^ We assumed that patients who failed treatment were not re-treated, and all patients completely complied with the treatment.

Costs and health outcomes were estimated in a lifetime period with 1-year cycle length. A within-cycle correction method, the Simpson’s one-third rule correction method,^29^ was applied, as suggested by Elbasha and Chhatwal.^30^ An annual discount rate of 3% was used for both costs and outcomes, as suggested by the World Health Organization.^31^ Costs were converted to Vietnamese Dong in the year 2019 using Vietnam’s consumer price index,^32^ then converted to US dollars using the exchange rate of $1.00 = 23 143 Vietnamese Dong.^33^ The cost-effectiveness threshold of 1 gross domestic product (GDP) per capita of Vietnam ($2389) was used.^34^ The model was designed and run in Microsoft Office Excel.

The CUA from a societal perspective estimated cost components such as direct medical and direct non-medical costs, including time cost associated with the treatment of patients and their caregivers (ie, informal care). The indirect costs (ie, morbidiy cost and mortality cost) were excluded to avoid a double-counting issue in the CUA.^35^^,^^36^ Meanwhile, the analysis from a payer perspective only estimated direct medical cost covered by the payer in Vietnam—the Vietnam Social Security.

### Interventions and Comparator

All DAA regimens in the current Vietnamese health insurance drug list,^14^ including SOF/LDV, SOF+DCV, and SOF/VEL, were compared with the old standard of HCV treatment (ie, PR). Recommendations of treatment regimens and durations were based on Vietnam’s current treatment guidelines.^21^^,^^22^

### Model Parameters

The model considered the following input parameters, classified into 4 major groups: transition probabilities, treatment efficacy, costs, and utilities (Table 1).

**Table 1:** Input parameters used in the model.

Input parameters		Mean	Standard error	Distribution	Source
Transition probabilities					
*From*	*To*				
CHC	CC	0.019	0.005	Beta	^38^
CC	DC	0.056	0.014	Beta	^37^
	HCC	0.056	0.014	Beta	^37^
DC	HCC	0.056	0.014	Beta	^37^
	LRD	0.151	0.038	Beta	^37^
HCC	LRD (year 1)	0.118	0.030	Beta	^38^
	LRD (from year 2)	0.222	0.056	Beta	^38^
SVR (CC)	HCC	0.018	0.005	Beta	^27^
Treatment efficacy (SVR12)					
*Genotype 1*					
SOF/LDV		0.980	0.008	Beta	Meta-analysis
SOF/VEL		0.980	0.005	Beta	Meta-analysis
SOF+DCV		0.990	0.020	Beta	Meta-analysis
PegIFN+RBV		0.625	0.096	Beta	^47^
*Genotype 6*					
SOF/LDV		0.992	0.008	Beta	^46^
SOF/VEL		1.000	-	Beta	^46^
SOF+DCV		0.990	0.020	Beta	^46^
PegIFN+RBV		0.802	0.027	Beta	^47^
Costs (US dollars, 2019)					
*Direct medical cost*					
*Drug cost*					
SOF/LDV (12-week)		1,384.4	276.9	gamma	Primary data
SOF/VEL (12-week)		1,739.7	347.9	gamma	Primary data
SOF+DCV (12-week)		1,733.0	346.6	gamma	Primary data
PegIFN (per week)		114.5	22.9	gamma	Primary data
RBV (per day)		1.2	0.2	gamma	Primary data
*Monitoring cost*					
DAAs (12-week)		355.6	71.1	gamma	Primary data
DAAs (24-week)		360.4	72.1	gamma	Primary data
PegIFN+RBV (48-week)		525.3	105.1	gamma	Primary data
*Cost of palliative care (per year)*					
CHC		108.5	21.7	gamma	Primary data
CC		598.7	119.7	gamma	Primary data
DC		964.1	192.8	gamma	Primary data
HCC		3,676.0	735.2	gamma	Primary data
*Direct non-medical cost (per year)*					
*For antiviral treatment*					
CHC treated with DAAs		87.6	17.5	gamma	Primary data
CC treated with DAAs		268.1	53.6	gamma	Primary data
CHC/CC treated with PegIFN+RBV		235.7	47.1	gamma	Primary data
*For palliative care*					
CHC		174.1	34.8	gamma	Primary data
CC		212.2	42.4	gamma	Primary data
DC		364.5	72.9	gamma	Primary data
HCC		334.2	66.8	gamma	Primary data
*Time cost (per year)*					
*For antiviral treatment*					
CHC treated with DAAs		129.2	25.8	gamma	Primary data
CC treated with DAAs		165.1	33.0	gamma	Primary data
CHC treated with PegIFN+RBV		129.2	25.8	gamma	Assumed
CC treated with PegIFN+RBV		165.1	33.0	gamma	Assumed
*For palliative care*					
CHC		189.8	38.0	gamma	Primary data
CC		201.6	40.3	gamma	Primary data
DC		369.9	74.0	gamma	Primary data
HCC		361.5	72.3	gamma	Primary data
Utilities					
CHC		0.878	0.026	triangular	Primary data
CC		0.695	0.077	triangular	Primary data
DC		0.491	0.155	triangular	Primary data
HCC		0.358	0.160	triangular	Primary data
Epidemiology					
*Genotype distribution in CHC-CC population (target population)*					
Genotype 1		0.358	0.072	Beta	^8^
Genotype 6		0.642	0.128	Beta	^8^
*CHC-CC distribution in genotype 1*					
CHC		0.550	0.110	Beta	^9^
CC		0.450	0.090	Beta	^9^
*CHC-CC distribution in genotype 6*					
CHC		0.620	0.124	Beta	^9^
CC		0.380	0.076	Beta	^9^

CC indicates compensated cirrhosis; CHC, non-cirrhotic chronic hepatitis C; DAA, direct-acting antiviral; DC, decompensated cirrhosis; DCV, daclatasvir; HCC, hepatocellular carcinoma; LDV, ledipasvir; LRD, liver-related death; PegIFN, pegylated-interferon; SOF, sofosbuvir; SVR12, sustained virologic response at 12th week after treatment; RBV, ribavirin; VEL, velpatasvir.

#### Transition probabilities

The disease-related transition probabilities were obtained from Japan owing to the unavailability of data in Vietnam. Among Asian countries, Japan was found to have the most comprehensive set of transition probabilities estimated from their own population.^26^^,^^27^^,^^37^^,^^38^ To test the validity of these transition probabilities, cross-model validations were performed to compare the long-term disease progression predicted by this model against other published models. The age-specific probabilities of all-causes mortality were derived from the latest life table of Vietnam published by the Vietnam General Statistics Office in 2016.^28^

#### Treatment efficacy

Treatment efficacy of HCV antivirals was measured by the rate of SVR at the 12th week after stopping antivirals (SVR12). For HCV genotype 1, a systematic search of existing meta-analyses on the efficacy of DAA regimens in genotype 1 was performed, which identified 7 relevant meta-analyses.^39^, ^40^, ^41^, ^42^, ^43^, ^44^, ^45^ Individual trials were extracted from these meta-analyses by DAA regimens. For regimens that had only 1 meta-analysis (such as SOF/VEL), we used the result of that meta-analysis; meanwhile, for regimens that had more than 1 meta-analysis (such as SOF/LDV and SOF+DCV), we performed a new pooling and used our pooled result. Regarding HCV genotype 6, we conducted a meta-analysis to pool SVR12 from existing trials on DAA regimens in genotype 6.^46^ The treatment efficacy of PR was obtained from a published meta-analysis.^47^

#### Costs

Direct medical, direct non-medical, and time costs associated with treatment were obtained from primary data collection in Vietnam’s 2 central-level hospitals, Bach Mai Hospital and the National Hospital of Tropical Diseases, in 2019. Ethics approval for the study was granted by the Institutional Review Board of the Hanoi University of Public Health, Vietnam, on July 24, 2018.^48^

Direct medical costs included costs of antivirals (SOF/LDV, SOF/VEL, SOF+DCV, PR), treatment monitoring (HCV RNA test, fibroscan, ultrasound, genotyping, and other blood tests**)**, and palliative care for patients who failed to achieve SVR12**.** Direct medical costs were estimated by applying a cost**-**at**-**charge approach. In costing, the usual practice is to assume that market price was a reasonable approximation of monetary cost.^49^, ^50^, ^51^ Direct medical costs were collected retrospectively from patient records.

Direct non-medical costs included travel, accommodation, meals, and other relevant non-medical costs **(**eg**,** buying personal belongings for patients during hospitalization**)** of patients and their caregivers. These costs were estimated by prospectively interviewing patients or their caregivers**.**

Time costs were the costs of time loss associated with the treatment, which were borne by patients **(**or their caregivers**)** in seeking care and waiting and receiving care at hospital **(**or aiding patients to do so**).** These time costs were valuated using the human capital approach^35^ (ie, multiplying the days lost by the average daily wage)**.** The number of days lost were prospectively collected from interviewing patients or their caregivers**.**

#### Utilities

The health outcome of choice was quality-adjusted life-years (QALYs), which is the multiplication of life years (LYs) by utility score. The utility of each health state was obtained from primary data collection using the EQ-5D-5L questionnaire^52^^,^^53^ in the aforementioned hospitals in 2019, and the EQ-5D-5L value set of Vietnam was applied.^54^ Permission from the EuroQoL group was granted for using the Vietnamese version of the questionnaire. Ethics approval for the study was granted by the Institutional Review Board of the Hanoi University of Public Health.^48^ Each patient participated in 1 interview, conducted when the patient was at the hospital for either outpatient or inpatient care due to their HCV-related complications.

### Result Presentation

Total costs, LYs, and QALYs for each treatment were estimated in a lifetime period. In addition, lifetime cumulative incidence of HCV-related complications was calculated by cumulatively adding up the number of HCV-related complications from the first cycle to the last cycle in the model.

To estimate the cost-effectiveness of each regimen compared with PR, an incremental cost-effectiveness ratio, calculated by an incremental cost divided by an incremental LY or QALY, was estimated and compared with the cost-effectiveness threshold of 1 GDP per capita of Vietnam per QALY gained. Furthermore, the corresponding net monetary benefit (NMB) of all 3 DAA regimens was compared in order to rank their cost-effectiveness.

### Uncertainty Analysis

#### Parameter uncertainty

To test the robustness of the base-case results, a deterministic 1-way sensitivity analysis was performed by stochastically varying 1 parameter at a time between its lower and upper limits, and the corresponding change of NMB was captured and shown graphically as a tornado diagram. Key parameters varied including discount rates (±3%), transition probabilities (±20%), treatment efficacies (because the SVR12s of DAAs were relatively high, their range for variation was purposely set between –20% and 100%), costs (±20%), utilities (95% confidence interval [CI]), and epidemiology parameters (±20%).

In addition, to assess the impact of parameter uncertainty on the results, probabilistic sensitivity analyses were conducted by stochastically varying all key parameters simultaneously within their probability distribution. The Monte Carlo simulation was run in 1000 iterations, then shown graphically as a cost-effectiveness plane and a cost-effectiveness acceptability curve.

#### Scenario analysis

In the base-case CUA, the government’s co-payment rate for DAA regimens was set at 50%, according to current regulation.^14^ To examine the generalizability of the model, scenarios of different government’s copayment rates for DAA regimens (50%, 70%, 90%, and 100%) were also explored.

### Value of Information Analysis

The study performed the value of information analysis in terms of total expected value of perfect information (EVPI) and expected value of partial perfect information (EVPPI) in order to estimate the expected value of further information to resolve the current uncertainty and to identify which type of information would be the most worthwhile.

The time horizon for estimating EVPI for population was set at 5 years, which was assumed to be the lifespan of these DAA regimens. The annual affected population was calculated by multiplying the prevalent number of CHC and CC patients with genotypes 1 and 6^1^^,^^8^ with the current diagnosis rate,^55^ treatment coverage,^55^ and proportion of eligible-to-treat population^22^ (Appendix Table 1 in Supplemental Materials found at https://doi.org/10.1016/j.jval.2020.03.018). Consequently, the 5-year affected population, given the annual discount rate of 3%, was 3711 persons.

Both *pairwise* EVPI (each DAA compared with PR) and *general* EVPI (all DAAs and PR compared with each other) were estimated. The EVPI was estimated by different ceiling ratios, whereas the EVPPI used the ceiling ratios of 1 GDP per capita of Vietnam and was run by 100 outer loops and 100 inner loops.

## Results

### Model Validation

In addition to face validation performed by clinical experts, our model’s predictions of HCV natural history were cross-validated with other published modeling studies^56^, ^57^, ^58^, ^59^, ^60^, ^61^ (Fig. S1B in Supplemental Materials found at https://doi.org/10.1016/j.jval.2020.03.018). Regarding the cumulative incidence of CC (Fig. S1A), our study predicted a 20-year cumulative incidence of 29**.**1%, which was comparable with other studies,^57^, ^58^, ^59^^,^^61^ where this rate ranged from 27.0% to 29.1%. In addition, the 10-year cumulative incidence of DC, HCC, and LRD was predicted in our study, which were 15.5***%***, 18.1***%***, and 17.4***%,*** respectively. However, in comparison to the result of a multicenter follow-up study by van der Meer et al***,***^56^ our predicted rates were lower, and only the 10-year cumulative incidence of HCC was within the reported confidence limits in van der Meer’s study**.** These differences may be explained by the differences in baseline population between models—our model included HCV patients at mild to severe fibrosis stage, whereas in van der Meer’s model, all patients were at severe fibrosis stage.^56^

### Base-Case Analysis

The lifetime cumulative new cases of HCV-related complications (ie, DC, HCC, LRD) were projected in the model. Compared with PR, all DAA regimens were associated with lower cumulative incidence of all complications. Specifically, DAA regimens decreased the cumulative incidence of DC by 95.3% to 97.1%, whereas the incidence of HCC and LRD was reduced by 23.5% to 23.9% and by 39.8% to 40.6%, respectively. In all cases, SOF/VEL always resulted in the lowest incidence, thereby demonstrating the highest effectiveness in terms of avoiding HCV-related complications (Appendix Table 2 in Supplemental Materials found at https://doi.org/10.1016/j.jval.2020.03.018)*.*

The base-case cost-effectiveness analysis indicated that all DAA regimens dominated PR (ie, were less costly and more effective), based on both societal and payer perspectives (Table 2). From a societal perspective, compared with PR, treatments with DAA regimens in Vietnam’s HCV population genotypes 1 and 6 were associated with the increment of overall life expectancy by 0.65 to 0.66 years and the increment of discounted QALYs by 1.33 to 1.35 QALYs, whereas costs were significantly decreased by $6519 to $7246. In addition, the lifetime costs of DAA regimens and PR were further classified into intervention cost (ie, costs of drugs and treatment monitoring) and non-intervention cost (ie, costs of palliative care) and shown graphically, which demonstrated the lower values of both intervention and non-intervention costs of DAA regimens compared with PR (Fig. S2 in Supplemental Materials found at https://doi.org/10.1016/j.jval.2020.03.018).

**Table 2:** Cost-effectiveness of direct-acting antivirals compared with pegylated-interferon plus ribavirin for treatment of chronic hepatitis C virus genotypes 1 and 6 in Vietnam (US dollars, 2019).

Societal perspective	PR	SOF/LDV	SOF/VEL	SOF+DCV
Discounted cost	11 301	4430	4055	4782
Discounted LYs	14.69	15.34	15.36	15.35
Discounted QALYs	13.74	15.06	15.09	15.08
Incremental cost		–6870	–7246	–6519
Incremental LYs		0.65	0.66	0.66
Incremental QALYs		1.33	1.35	1.34
ICER per LY		Dominant	Dominant	Dominant
ICER per QALY		Dominant	Dominant	Dominant
NMB∗	21 519	31 555	31 993	31 234

Payer perspective	PR	SOF/LDV	SOF/VEL	SOF+DCV
Discounted cost	4611	2317	2101	2461
Discounted LYs	14.69	15.34	15.36	15.35
Discounted QALYs	13.74	15.06	15.09	15.08
Incremental cost		–2294	–2509	–2149
Incremental LYs		0.65	0.66	0.66
Incremental QALYs		1.33	1.35	1.34
ICER per LY		Dominant	Dominant	Dominant
ICER per QALY		Dominant	Dominant	Dominant
NMB∗	28 209	33 669	33 947	33 555

DAA indicates direct-acting antiviral; DCV, daclatasvir; GDP, gross domestic product; ICER, incremental cost-effectiveness ratio; LDV, ledipasvir; LY, life-year; NMB, net monetary benefit; PR, pegylated-interferon plus ribavirin; SOF, sofosbuvir; VEL, velpatasvir; QALY, quality-adjusted life-year

∗ At cost-effectiveness threshold of 1 GDP per capita in Vietnam: $2389 per QALY gained^34^

Among 3 DAA regimens, SOF/VEL was associated with the lowest lifetime cost at $4055 per patient and was found to be the most efficacious at 15.09 QALYs. Furthermore, in order to directly compare the cost-effectiveness among the 3 DAA regimens, the corresponding NMB of all 3 DAA regimens was calculated, which showed that SOF/VEL was the most cost-effective regimen, followed by SOF/LDV as the second, and SOF+DCV as the third, from both perspectives (Table 2).

Different scenarios of government’s co-payment rates (50%-100%) were explored. In all scenarios, 3 DAAs remained cost-saving in comparison to PR, and SOF/VEL remained the most cost-effective regimen, regardless of the copayment rates, from both perspectives (Appendix Table 3 in Supplemental Materials found at https://doi.org/10.1016/j.jval.2020.03.018).

### Parameter Uncertainty

**Figure 2:**
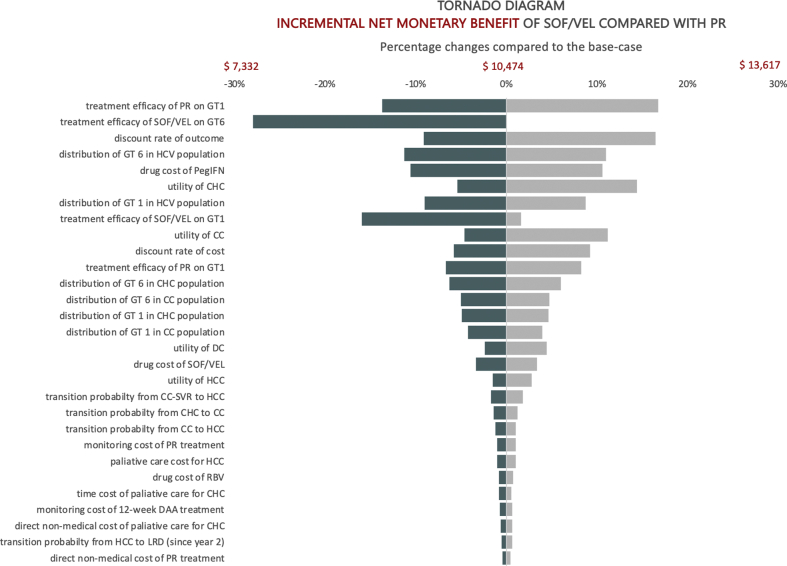
Tornado diagram of sofosbuvir/velpatasvir compared with pegylated-interferon plus ribavirin for treatment of chronic hepatitis C virus genotypes 1 and 6 in Vietnam from a societal perspective (US dollars, 2019).

One-way deterministic sensitivity analysis was shown graphically as a tornado diagram (Fig. 2), which illustrated the parameters that most heavily influenced the incremental NMB (at the cost-effectiveness threshold of 1 GDP per capita of Vietnam) of the most cost-saving regimen (ie, SOF/VEL) compared to PR, from a societal perspective. The incremental NMB was always positive, which indicated that SOF/VEL always remained cost-effective at the threshold of 1 GDP per capita of Vietnam. In addition, the incremental NMB was most sensitive to the treatment efficacy of PR and SOF/VEL, the discount rates for outcome and cost, the distribution of HCV genotype 1 and 6 in Vietnam, and the utility values. Furthermore, discount rates for costs at 3%, QALY at 1.5%, and 1.5% for both QALY and costs were tested, which confirmed the base-case results (Appendix Table 4 in Supplemental Materials found at https://doi.org/10.1016/j.jval.2020.03.018).

CC indicates compensated cirrhosis; CHC, non-cirrhotic chronic hepatitis C; DAA, direct-acting antiviral; DC, decompensated cirrhosis; DCV, daclatasvir; GT, genotype; HCC, hepatocellular carcinoma; HCV, hepatitis C virus; LDV, ledipasvir; LRD, liver-related death; PegIFN, pegylated-interferon; PR, pegylated-interferon + ribavirin; RBV, ribavirin; SOF, sofosbuvir; SVR, sustained virologic response; RBV, ribavirin; VEL, velpatasvir.

**Figure 3:**
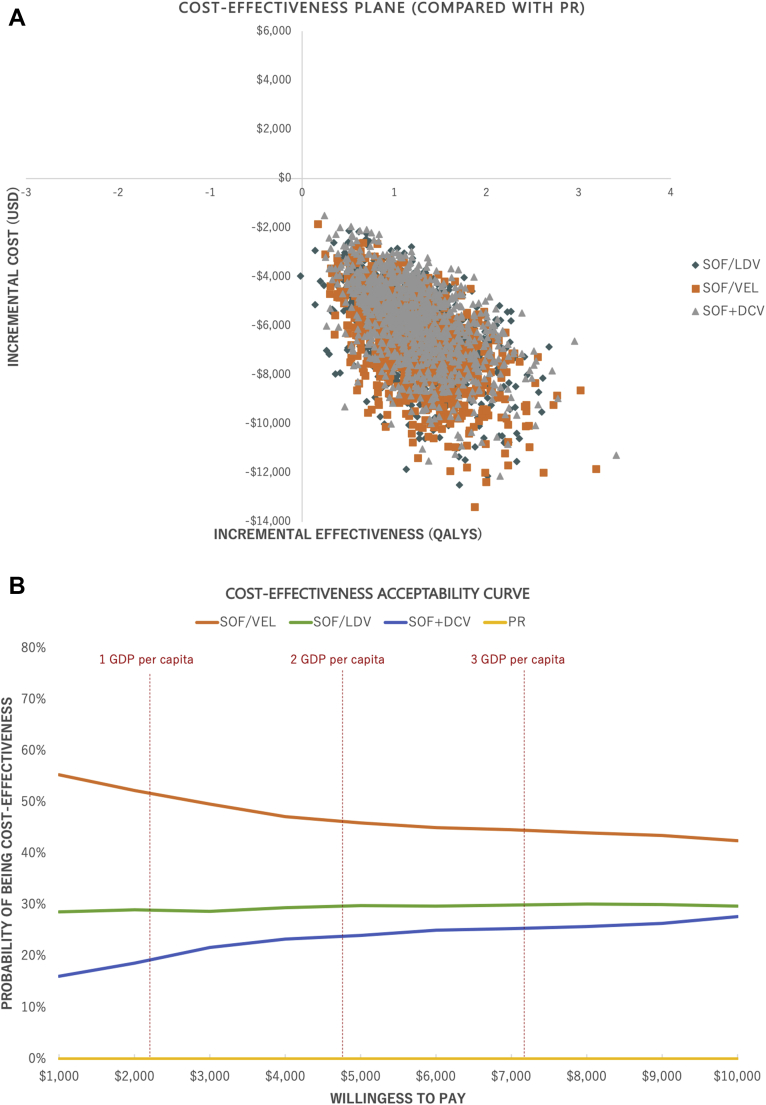
Probabilistic sensitivity analysis of DAAs compared with pegylated-interferon plus ribavirin for treatment of chronic hepatitis C virus genotype 1 and 6 in Vietnam, under societal perspective (US dollars, 2019).

The probabilistic sensitivity analysis, performed from a societal perspective, confirmed the robustness of the base-case cost-effectiveness result, of which all DAA regimens dominated PR, and SOF/VEL was the most cost-saving regimen. Specifically, the cost-effectiveness plane showed the cost-saving result of DAA regimens in 100% of the 1000 simulations, whereas the cost-effectiveness acceptability curve indicated that at all willingness-to-pay thresholds, SOF/VEL always remained the most cost-effective regimen (Fig. 3).

DAA indicates direct-acting antiviral; DCV, daclatasvir; GDP, gross domestic product; LDV, ledipasvir; PR, pegylated-interferon plus ribavirin; QALY, quality-adjusted life-year; SOF, sofosbuvir; VEL, velpatasvir.

### Value of Information Analysis

**Figure 4:**
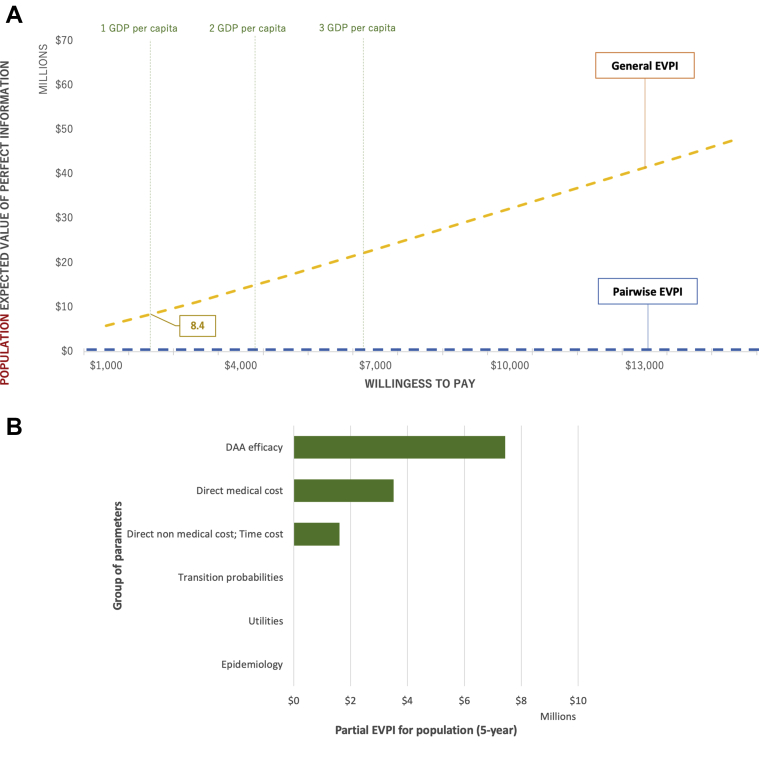
Value of information analysis (US dollars, 2019).

The pairwise EVPI for population (each DAA regimen compared to PR) was zero at all willingness-to-pay values (Fig. 4), suggesting that the cost-saving results of any DAA regimen compared to PR were robust and not likely to change even under perfect information; therefore, further information might not be necessary in this case.

DAA indicates direct-acting antiviral; EVPI, expected value of perfect information; EVPPI, expected value of partial perfect information; GDP, gross domestic product.

The general EVPI for population (3 DAA regimens and PR compared to each other) was $8.4 million at the willingness to pay of 1 GDP per capita of Vietnam (Fig. 4), corresponding to an individual EVPI of $480. The difference between pairwise EVPI and general EVPI implied that there were uncertainties among DAA regimens (but not between any DAA regimen versus PR), which were worthwhile to eliminate. As the willingness to pay increased, the EVPI become higher owing to the increment of NMB; however, no peak of EVPI curve could be observed, suggesting that the current cost-effectiveness ranking among DAA regimens was robust and not likely to change, even under the perfect information.

The 5-year general EVPPI at the willingness to pay of 1 GDP per capita of Vietnam was estimated on all major parameter groups, including DAAs’ treatment efficacy, direct medical cost, direct non-medical and time cost, transition probabilities, utilities, and epidemiology (Fig. 4). The uncertainties were observed in DAA efficacy and cost parameter groups, which suggested that if further research is conducted, researchers should aim to obtain better data on relative treatment efficacy between DAA regimens or better cost estimation.

## Discussion

In this study, the cost-effectiveness of all available DAA regimens in the Vietnamese health insurance drug list^14^ for HCV patients with genotypes 1 and 6 was assessed. All DAA regimens were associated with lower cost and higher effectiveness, thereby being cost-saving compared with PR, from both societal and payer perspectives. Our results may be particularly useful for countries with a high prevalence of HCV genotype 6, such as Laos (95.6%), Cambodia (56.0%), Myanmar (49.0%), and Thailand (21.8%).^12^

The robustness of these findings was tested by performing extensive deterministic and probabilistic sensitivity analyses, which found that DAAs always remained cost-saving despite all key parameters being varied across wide yet plausible ranges. The most influential parameters were similar to those reported by other modeling studies in the field.^16^

In addition, the result of EVPI for the population indicated a small degree of uncertainty in the CUA results in all willingness-to-pay scenarios, and the decision to adopt DAA regimens would not be likely to change even under perfect information. Nevertheless, if policy makers demand more certain recommendation in terms of cost-effectiveness ranking among DAA regimens, further research to obtain better data on relative treatment efficacies among DAA regimens or better cost estimation might be worthwhile.

The cost-saving results of DAA regimens in Vietnam were mainly attributed to the significantly lower DAA prices in Vietnam compared with their original prices. The reduced prices were due to the result of voluntary license agreements signed by the originator companies, which allowed HCV patients to access generic DAAs. Currently, more than 100 countries around the world have benefited from the license agreements, including a majority of LMICs.^62^ Our study findings are in accordance with several studies conducted in Asian countries, including a study of Rattanavipapong^63^ in Thailand, where SOF/LDV and SOF+DCV regimens were cost-saving compared with PR, and another study of Igarashi^64^ in Japan, where SOF/LDV was also found to be cost-saving compared with PR. Some other studies that compared DAA regimens to no treatment also reported the cost-saving result of DAA regimens, including a study of Aggarwal^65^ in India on SOF/LDV and SOF+DCV, and another study of Goel^66^ in India on SOF/VEL.

To meet the World Health Organization’s goal of eliminating HCV as a public health threat by 2030,^67^ a rapid scale-up of DAA treatment is required. Nevertheless, despite demonstrating the cost-saving benefits for the society and payer in Vietnam, scale-up of DAA treatment in reality might depend on other factors, such as enabling financial accessibility for the HCV population, which is among the most important factors. In a study by Thu Nguyen et al^68^ that measured the willingness to pay of HCV patients for diagnosis tests and 12-week antiviral treatment, 54.6% of patients were not willing to pay more than $440. Meanwhile, our model estimated that the total cost for diagnosis tests and the 12-week DAA treatment was at least $980; in this case the DAA regimen used was the cheapest one (ie, SOF/LDV), the patient had health insurance (ie, the patient only needed to pay for 50% of DAA cost and an average of 20% of testing cost), and the health state was non-cirrhotic. Hence, the government should consider strategies to reduce patients’ out-of-pocket payments for DAA treatment. Several strategies might be considered, such as: (1) implementing further price-reduction strategies; (2) increasing the government’s copayment rate for DAA regimens, which currently is 50% (it should be noted that, DAA regimens are still cost-saving at 100% the government’s copayment rate, as demonstrated in the scenario analysis); or (3) seeking donor support.

### Limitations

This study has several limitations. First, our model assumed that all patients complied with the treatment, although dropouts were likely to occur in reality, which may overestimate the benefits of DAA regimens. Second, we assumed that patients had no comorbidities, but in reality, comorbidities may be common at 50 years old; therefore, we may underestimate the benefits of DAA regimens. Third, we did not consider the benefits of HCV treatments in preventing transmissions in society; however, it is expected that the cost-effectiveness results of DAA regimens would be more favorable if this public health benefit were taken into account. Fourth, our model did not include liver transplantation as a health state because it was neither included in the health insurance package nor currently widely accessible in Vietnam. Lastly, owing to the unavailability of some epidemiological data in Vietnam, such as annual age-specific incidence and prevalence of HCV and its complications, in addition to the lack of longitudinal studies on HCV patients, we cannot perform the model calibration to fit actual data in the Vietnamese context. However, the results of all uncertainty analyses have confirmed the robustness of our cost-effectiveness results.

## Conclusions

In conclusion, this study has demonstrated that all 3 DAA regimens in the Vietnamese health insurance drug list (ie, SOF/LDV, SOF+DCV, SOF/VEL) are cost-saving in HCV patients with genotypes 1 and 6 from both societal and payer perspectives; thus, allocating resources for DAA treatment is surely a rewarding public health investment. Our results may be particularly useful for countries with a high prevalence of HCV genotype 6. Furthermore, because more current evidence suggesting that DAAs might be effective for advanced-stage HCV patients,^69^^,^^70^ future studies in LMICs should investigate the cost-utility of DAAs among patients at all disease states.

In the DAAs era, the elimination of HCV as a public health threat would be feasible, as illustrated by the evidence that we have presented, yet this could not be accomplished without seamless political commitments, a responsive health system, and strong public support.

## Figures and Tables

**Figure S1A:**
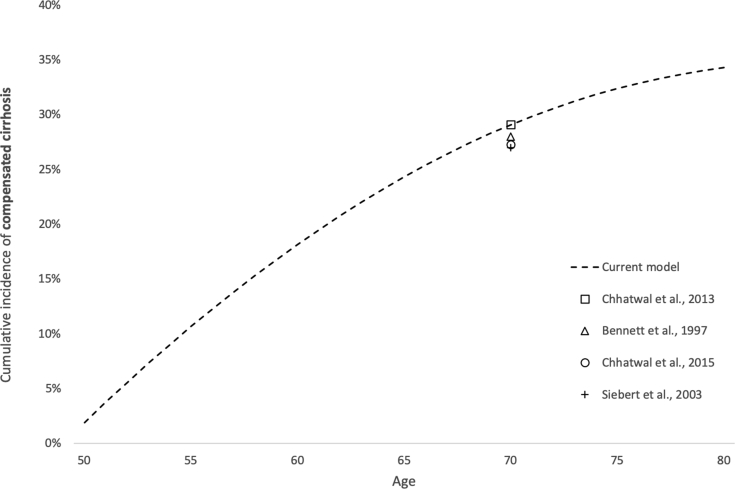


**Figure S1B:**
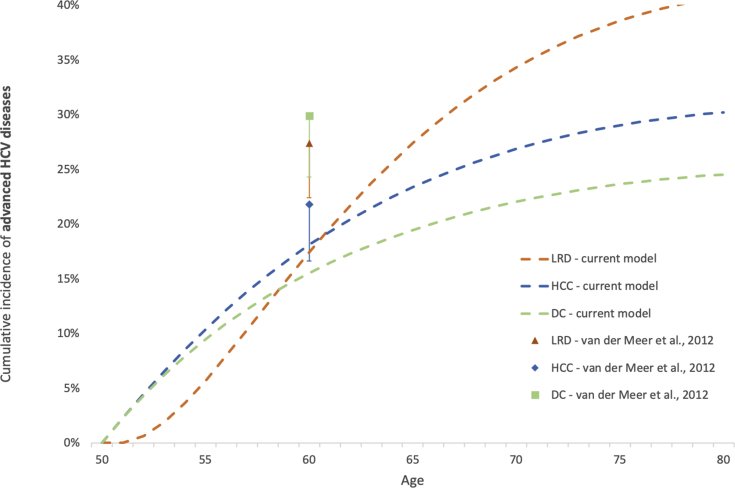


**Figure S2:**
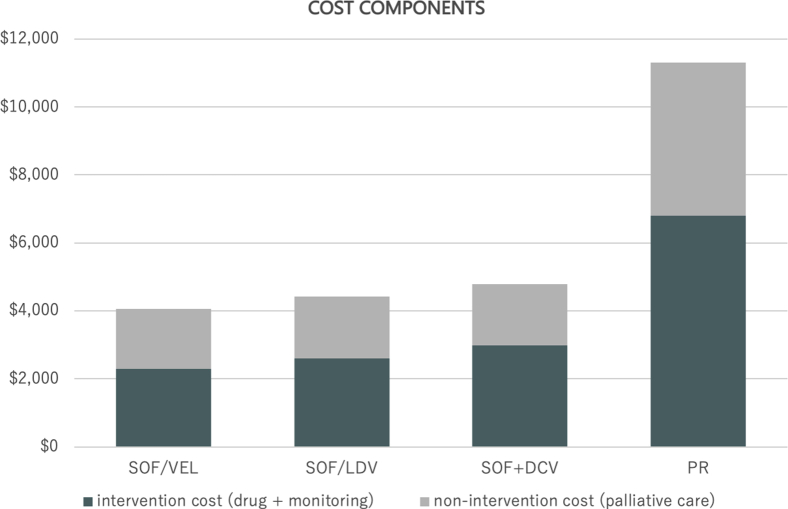


## References

[1] Nguyen T.V.T., Tran D.Q., Nguyen T.A. (2018). Estimate and projection of disease burden and investment case for hepatitis C in Vietnam. J Viral Hepat.

[2] Gish R.G., Bui T.D., Nguyen C.T. (2012). Liver disease in Vietnam: screening, surveillance, management and education: a 5-year plan and call to action. J Gastroenterol Hepatol.

[3] Kakumu S., Sato K., Morishita T. (1998). Prevalence of hepatitis B, hepatitis C, and GB virus C/hepatitis G virus infections in liver disease patients and inhabitants in Ho Chi Minh. Vietnam. J Med Virol.

[4] Huy T.T.T., Hiroshi U., Quang X.V. (2003). Prevalence of hepatitis virus types B through E and genotypic distribution of HBV and HCV in Ho Chi Minh City. Vietnam. Hepatol Res.

[5] Nguyen T.T.V., McLaws M.L., Dore G. (2007). Prevalence and risk factors for hepatitis C infection in rural North Vietnam. Hepatol Int.

[6] World Health Organization (2017). *Global Hepatitis Report 2017.* Geneva, Switzerland: World Health Organization.

[7] Pham D.A., Leuangwutiwong P., Jittmittraphap A. (2009). High prevalence of hepatitis C virus genotype 6 in Vietnam. Asian Pac J Allergy Immunol.

[8] Van H.P., Huyen D.P.N., Phat T.H. (2011). Very high prevalence of hepatitis C virus genotype 6 variants in southern Vietnam: Large-scale survey based on sequence determination. Jpn J Infect Dis.

[9] Thuy P.T.T., Dat H.T., Toan N.B. (2014). The different incidences of HCV genotypes using two different regions of classification: 5'NC and NS5B in Vietnamese patients at Medic Medical Center. Ho Chi Minh City: Medic Medical Center.

[10] Dat H.T., Thuy P.T.T., Tong N.T. (2006). Prevalence of HCV genotypes in Vietnam. Ho Chi Minh City J of Medicine.

[11] Quang V.M., Phong N.D., Khiem D.T. (2009). Epidemiological, clinical and paraclinical characteristics of viral hepatitis C patients treated at Ho Chi Minh City Hospital of Tropical Diseases in 2006-2007. Ho Chi Minh City J of Medicine.

[12] Gower E., Estes C., Blach S., Razavi-Shearer K., Razavi H. (2014). Global epidemiology and genotype distribution of the hepatitis C virus infection. J Hepatol.

[13] Thrift A.P., El-Serag H.B., Kanwal F. (2017). Global epidemiology and burden of HCV infection and HCV-related disease. Nat Rev Gastroenterol Hepatol.

[14] Vietnam Ministry of Health Promulgating the List of modern medicines, biologicals, radiopharmaceuticals and tracers covered by health insurance, insurance coverage ratio and payment conditions thereof. Circular No. 30/2018/TT-BYT. https://thuvienphapluat.vn/van-ban/Bao-hiem/Thong-tu-30-2018-TT-BYT-thanh-toan-thuoc-hoa-duoc-sinh-pham-cua-nguoi-tham-gia-bao-hiem-y-te-400326.aspx.

[15] He T., Lopez-Olivo M.A., Hur C., Chhatwal J. (2017). Systematic review: cost-effectiveness of direct-acting antivirals for treatment of hepatitis C genotypes 2-6. Aliment Pharmacol Ther.

[16] Chhatwal J., He T., Lopez-Olivo M.A. (2016). Systematic review of modelling approaches for the cost effectiveness of hepatitis C treatment with direct-acting antivirals. Pharmacoeconomics.

[17] Chhatwal J., He T., Hur C., Lopez-Olivo M.A. (2017). Direct-acting antiviral agents for patients with hepatitis C virus genotype 1 infection are cost-saving. Clin Gastroenterol Hepatol.

[18] Puig-Junoy J., Pascual-Argente N., Puig-Codina L., Planellas L., Solozabal M. (2018). Cost-utility analysis of second-generation direct-acting antivirals for hepatitis C: a systematic review. Expert Rev Gastroenterol Hepatol.

[19] Do S.H., Yamada H., Fujimoto M. (2015). High prevalences of hepatitis B and C virus infections among adults living in Binh Thuan Province. Vietnam. Hepatol Res.

[20] Ngoc C.L., Thanh T.T.T., Lan P.T.T. (2019). Differential prevalence and geographic distribution of hepatitis C virus genotypes in acute and chronic hepatitis C patients in Vietnam. PLoS One.

[21] Vietnam Ministry of Health. The Treatment Guideline in Diagnosis and Treatment of Hepatitis C. Decision No 4817/QD-BYT. https://thuvienphapluat.vn/van-ban/the-thao-y-te/quyet-dinh-4817-qd-byt-nam-2013-tai-lieu-huong-dan-chan-doan-dieu-tri-viem-gan-vi-rut-c-215782.aspx. Accessed 10 July 2020.

[22] Vietnam Ministry of Health. The Treatment Guideline in Diagnosis and Treatment of Hepatitis C. Decision No 5012/QD-BYT. https://thuvienphapluat.vn/van-ban/the-thao-y-te/Quyet-dinh-5012-QD-BYT-chan-doan-dieu-tri-benh-viem-gan-vi-rut-c-2016-322718.aspx. Accessed 10 July 2020.

[23] Kapol N., Lochid-amnuay S., Teerawattananon Y. (2016). Economic evaluation of pegylated interferon plus ribavirin for treatment of chronic hepatitis C in Thailand: genotype 1 and 6. BMC Gastroenterol.

[24] Yoshida H., Shiratori Y., Moriyama M. (1999). Interferon therapy reduces the risk for hepatocellular carcinoma: national surveillance program of cirrhotic and noncirrhotic patients with chronic hepatitis C in Japan: inhibition of hepatocarcinogenesis by interferon therapy. Ann Intern Med.

[25] Tateyama M., Yatsuhashi H., Taura N. (2011). Alpha-fetoprotein above normal levels as a risk factor for the development of hepatocellular carcinoma in patients infected with hepatitis C virus. J Gastroenterol.

[26] Maruoka D., Imazeki F., Arai M., Kanda T., Fujiwara K., Yokosuka O. (2012). Long–term cohort study of chronic hepatitis C according to interferon efficacy. J Gastroenterol Hepatol.

[27] McEwan P., Ward T., Webster S. (2014). Estimating the long-term clinical and economic outcomes of daclatasvir plus asunaprevir in difficult-to-treat Japanese patients chronically infected with hepatitis C genotype 1b. Value Health Reg Issues.

[28] Vietnam (2016). Major findings: the 1/4/2015 Time-Point Population Change and Family Planning Survey. Hanoi, Vietnam: Statistical Publishing House.

[29] Davis P.J., Rabinowitz P. (1984). Methods of Numerical Integration. 2nd ed.

[30] Elbasha E.H., Chhatwal J. (2016). Myths and misconceptions of within-cycle correction: a guide for modelers and decision makers. Pharmacoeconomics.

[31] World Health Organization (2003). Making Choices in Health: WHO Guide to Cost-Effectiveness Analysis. Geneva.

[32] Tổng cục Thống kê Việt Nam (2019). Consumer Price index, Gold and USD Price Indexes, Whole Country: 2014-2019.

[33] Vietnam’s Ministry of Finance Announcement of monthly exchange rate. https://www.mof.gov.vn/webcenter/portal/btc/r/dn/tght/tght_chitiet?dDocName=MOFUCM168289&_afrLoop=89599587225073732.

[34] Tổng cục Thống kê Việt Nam (2017). National Accounts, State Budget and Insurance. Statistical Yearbook of Vietnam.

[35] Gold M.R., Siegel J.E., Russell L.B., Weinstein M.C. (1996). Cost-Effectiveness in Health and Medicine.

[36] Muennig P., Bounthavon M. (2016). Cost-Effectiveness Analyses in Health: A Practical Approach.

[37] Suga M. (2014). Development of the fundamental model for HCV: research on health-economics of various initiatives related to viral liver diseases. Ministry of Health, Labour and Welfare, Japan.

[38] Ishida H., Yotsuyanagi H. (2013). Study of the cost-effectiveness of the standard treatment for chronic hepatitis C. https://mhlw-grants.niph.go.jp/niph/search/Download.do?nendo=2013&jigyoId=135013&bunkenNo=201333004A&pdf=201333004A0005.pdf.

[39] Ahmed H., Abushouk A.I., Attia A. (2018). Safety and efficacy of sofosbuvir plus velpatasvir with or without ribavirin for chronic hepatitis C virus infection: a systematic review and meta-analysis. J Infect Public Health.

[40] Ahmed H., Abushouk A.I., Menshawy A. (2018). Meta-analysis of grazoprevir plus elbasvir for treatment of hepatitis C virus genotype 1 infection. Ann Hepatol.

[41] Ahmed H., Elgebaly A., Abushouk A.I., Hammad A.M., Attia A., Negida A. (2017). Safety and efficacy of sofosbuvir plus ledipasvir with and without ribavirin for chronic HCV genotype-1 infection: a systematic review and meta-analysis. Antivir Ther.

[42] Ji F., Wei B., Yeo Y.H. (2018). Systematic review with meta-analysis: effectiveness and tolerability of interferon-free direct-acting antiviral regimens for chronic hepatitis C genotype 1 in routine clinical practice in Asia. Aliment Pharmacol Ther.

[43] Kowdley K.V., Sundaram V., Jeon C.Y. (2017). Eight weeks of ledipasvir/sofosbuvir is effective for selected patients with genotype 1 hepatitis C virus infection. Hepatology (Baltimore, Md).

[44] Ferreira V.L., Tonin F.S., Assis Jarek N.A., Ramires Y., Pontarolo R. (2017). Efficacy of interferon-free therapies for chronic hepatitis C: a systematic review of all randomized clinical trials. Clin Drug Investig.

[45] Rezaee-Zavareh M.S., Hesamizadeh K., Behnava B., Alavian S.M., Gholami-Fesharaki M., Sharafi H. (2017). Combination of ledipasvir and sofosbuvir for treatment of hepatitis C virus genotype 1 infection: systematic review and meta-analysis. Ann Hepatol.

[46] Due O.T., Chaikledkaew U., Genuino A.J.M., Sobhonslidsuk A., Thakkinstian A. (2019). Systematic review with meta-analysis: efficacy and safety of direct-acting antivirals for chronic hepatitis C genotypes 5 and 6. Biomed Res Int.

[47] Nguyen N.H., McCormack S.A., Vutien P. (2015). Meta-analysis: superior treatment response in Asian patients with hepatitis C virus genotype 6 versus genotype 1 with pegylated interferon and ribavirin. Intervirology.

[48] Hanoi University of Public Health (2018). Ethical Review Board for Biomedical Research. Decision no 389/2018/YTCC-HD3 on ethical approval for research involving human subject participation.

[49] Gray A. (2011). Applied Methods of Cost-Effectiveness Analysis in Health Care.

[50] Drummond M.F., Sculpher M.J., Torrance G.W. (2015). Methods for the Economic Evaluation of Health Care Programmes.

[51] Morris S., Devlin N., Parkin D., Spencer A., Hoboken N.J. (2012). Economic Analysis in Healthcare. 2nd.

[52] EuroQol Group (1990). EuroQol: a new facility for the measurement of health-related quality of life. Health Policy.

[53] Reenen M., Janssen B. (2015). *EQ-5D-5L User Guide: Basic Information on How to Use the EQ-5D-5L Instrument.* Rotterdam: EuroQol Research Foundation.

[54] Mai VQ, Minh HV, Sun S, et al. An EQ-5D-5L value set for Vietnam. Qual Life Res. 29(7):1923-1933. 10.1007/s11136-020-02469-7 PMC7295839 32221805

[55] Vietnam General Department of Preventive Medicine (2017). World Health Organization. *A Report of Hepatitis C Situation and Management Strategy in Vietnam.* Hanoi, Vietnam: General Department of Preventive Medicine.

[56] van der Meer A.J., Veldt B.J., Feld J.J. (2012). Association between sustained virological response and all-cause mortality among patients with chronic hepatitis C and advanced hepatic fibrosis. JAMA.

[57] Bennett W., Inoue Y., Beck J., Wong J., Pauker S., Davis G. (1997). Estimates of the cost-effectiveness of a single course of interferon-α 2b in patients with histologically mild chronic hepatitis C. Ann Intern Med.

[58] Chhatwal J., Ferrante S.A., Brass C. (2013). Cost-effectiveness of boceprevir in patients previously treated for chronic hepatitis C genotype 1 infection in the United States. Value Health.

[59] Chhatwal J., Kanwal F., Roberts M.S., Dunn M.A. (2015). Cost-effectiveness and budget impact of hepatitis C virus treatment with sofosbuvir and ledipasvir in the United States. Ann Intern Med.

[60] Petta S., Cabibbo G., Enea M. (2014). Cost-effectiveness of sofosbuvir-based triple therapy for untreated patients with genotype 1 chronic hepatitis C. Hepatology (Baltimore, Md).

[61] Siebert U., Sroczynski G., Rossol S. (2003). Cost effectiveness of peginterferon α-2b plus ribavirin versus interferon α-2b plus ribavirin for initial treatment of chronic hepatitis C. Gut.

[62] World Health Organization (2018). Progress Report on Access to Hepatitis C Treatment: Focus on Overcoming Barriers in Low- and Middle-Income Countries.

[63] Rattanavipapong W., Anothaisintawee T., Teerawattananon Y. (2018). Revisiting policy on chronic HCV treatment under the Thai Universal Health Coverage: An economic evaluation and budget impact analysis. PLoS One.

[64] Igarashi A., Tang W., Guerra I., Marié L., Cure S., Lopresti M. (2017). Cost–utility analysis of ledipasvir/sofosbuvir for the treatment of genotype 1 chronic hepatitis C in Japan. Curr Med Res Opin.

[65] Aggarwal R., Chen Q., Goel A. (2017). Cost-effectiveness of hepatitis C treatment using generic direct-acting antivirals available in India. PLoS One.

[66] Goel A., Chen Q., Chhatwal J., Aggarwal R. (2018). Cost-effectiveness of generic pan-genotypic sofosbuvir/velpatasvir versus genotype-dependent direct-acting antivirals for hepatitis C treatment. J Clin Exp Hepatol.

[67] World Health Organization (2016). Combating Hepatitis B and C to Reach Elimination by 2030. Geneva, Switzerland: World Health Organization.

[68] Nguyen T., Cao T., Nguyen H. (2018). Viral hepatitis C elimination strategy in Vietnam: willingness to pay for treatment among patients receiving antiretroviral therapy and methadone treatment. Paper presented at: 22nd International AIDS Conference. 2018.

[69] Foster G.R., Irving W.L., Cheung M.C. (2016). Impact of direct acting antiviral therapy in patients with chronic hepatitis C and decompensated cirrhosis. J Hepatol.

[70] Kamp W.M., Sellers C.M., Stein S., Lim J.K., Kim H.S. (2019). Impact of direct acting antivirals on survival in patients with chronic hepatitis C and hepatocellular carcinoma. Sci Rep.

