# Supplementary material for: Cost-Utility Analysis of Direct-Acting Antivirals for Treatment of Chronic Hepatitis C Genotype 1 and 6 in Vietnam
Source: Value Health. 2020 Sep;23(9):1180–90. doi: 10.1016/j.jval.2020.03.018 (PMC7491253; doi:10.1016/j.jval.2020.03.018)
Supplement: Supplemental Materials [file mmc1.docx]

**SUPPLEMENTARY MATERIALS**

**Figure S1.** Cross-model validation

*DC, decompensated cirrhosis; HCC, hepatocellular carcinoma; LRD, liver-related death*

1. Cumulative incidence of compensated cirrhosis.

1. Cumulative incidence of advanced HCV diseases.

**Figure S2.** Components of lifetime costs of DAAs and PR (US dollars, 2019)

*DAA, Direct-Acting Antiviral; SOF, sofosbuvir; VEL, velpatasvir; DCV, daclatasvir; LDV, ledipasvir; PR, pegylated-interferon + ribavirin.*

**Table S1.** Parameters used to estimate the affected population in value of information analysis

*HCV, hepatitis C virus; CHC, non-cirrhotic chronic hepatitis C; CC, compensated cirrhosis.*

|  | **Value** | **Source** |
| --- | --- | --- |
| **Prevalence of CHC-CC genotype 1 and 6 (target population)** | | |
| HCV prevalent population (CHC-CC) | 964,767 | ^1^ |
| Proportion of HCV genotype 1 and 6 | 84.8% | ^8^ |
| Prevalence of CHC-CC genotype 1 and 6 | **818,122** |  |
| **Population receiving antivirals** |  |  |
| Diagnosis rate | **8.1%** | ^55^ |
| Treatment coverage | **5.6%** | ^55^ |
| Proportion of eligible-to-treat population | **100%** | ^22^ |

**Table S2.** Lifetime cumulative incidence of HCV-related complications for HCV population receiving DAAs compared with PR in Vietnam

*DC, decompensated cirrhosis; HCC, hepatocellular carcinoma; LRD, liver-related death; DAA, Direct-Acting Antiviral; SOF, sofosbuvir; VEL, velpatasvir; DCV, daclatasvir; LDV, ledipasvir; PR, pegylated-interferon + ribavirin.*

| **Regimen** | **Lifetime cumulative incidence per 10,000 patients**  **(% reduction compared to PR)** | | |
| --- | --- | --- | --- |
|  | **DC** | **HCC** | **LRD** |
| **SOF/LDV** | 31 (95.3%) | 1401 (23.5%) | 1174 (39.8%) |
| **SOF/VEL** | 19 (97.1%) | 1392 (23.9%) | 1159 (40.6%) |
| **SOF+DCV** | 25 (96.2%) | 1397 (23.7%) | 1167 (40.2%) |
| **PR** | 665 | 1830 | 1951 |

**Table S3**. Incremental net monetary benefit (INB) of DAAs compared to PR for treatment of chronic HCV genotype 1 and 6 in Vietnam by analysis perspective (US dollars, 2019).

**Willingness-to-pay threshold:** one GDP per capita of Vietnam: 2,389 US dollars per QALY gained ^34^

*DAA, Direct-Acting Antiviral; SOF, sofosbuvir; VEL, velpatasvir; DCV, daclatasvir; LDV, ledipasvir; PR, pegylated-interferon + ribavirin; LY, Life-Year; QALY, Quality-Adjusted Life-Year; INB, Incremental net monetary benefit.*

| **Government’s co-payment rate for DAAs** | **Treatment regimens** | **INB by QALYs gained** | |
| --- | --- | --- | --- |
|  |  | Societal perspective | Payer perspective |
| 50% (base-case) | SOF/VEL | 10,474 | 5,738 |
|  | SOF/LDV | 10,036 | 5,460 |
|  | SOF+DCV | 9,716 | 5,346 |
|  | PR | Reference | Reference |
| 70% | SOF/VEL | 10,474 | 5,390 |
|  | SOF/LDV | 10,036 | 5,071 |
|  | SOF+DCV | 9,716 | 4,859 |
|  | PR | Reference | Reference |
| 90% | SOF/VEL | 10,474 | 5,042 |
|  | SOF/LDV | 10,036 | 4,681 |
|  | SOF+DCV | 9,716 | 4,372 |
|  | PR | Reference | Reference |
| 100% | SOF/VEL | 10,474 | 4,868 |
|  | SOF/LDV | 10,036 | 4,487 |
|  | SOF+DCV | 9,716 | 4,128 |
|  | PR | Reference | Reference |

**Table S4**. Deterministic sensitivity analysis on discount rates for costs and outcomes: Cost-effectiveness of DAAs compared with PR for treatment of chronic HCV genotype 1 and 6 in Vietnam (US dollars, 2019)

*DAA, Direct-Acting Antiviral; SOF, sofosbuvir; VEL, velpatasvir; DCV, daclatasvir; LDV, ledipasvir; PR, pegylated-interferon + ribavirin; LY, Life-Year; QALY, Quality-Adjusted Life-Year; INB, Incremental net monetary benefit.*

**Scenario 1. Discount rates at 3.0% for costs and 1.5% for outcomes**

1. *Societal perspective*

|  | **PR** | | **SOF/LDV** | **SOF/VEL** | | **SOF+DCV** | |  |
| --- | --- | --- | --- | --- | --- | --- | --- | --- |
| Discounted cost | 11,301 | | 4,430 | 4,055 | | 4,782 | |  |
| Discounted LYs | 17.32 | | 18.17 | 18.19 | | 18.18 | |  |
| Discounted QALYs | 16.20 | | 17.83 | 17.87 | | 17.85 | |  |
| Incremental cost |  | -6,870 | | | -7,246 | | -6,519 | |
| Incremental LYs |  | 0.85 | | | 0.87 | | 0.86 | |
| Incremental QALYs |  | 1.63 | | | 1.66 | | 1.65 | |
| ICER per LY |  | | Dominant | Dominant | | Dominant | |  |
| ICER per QALY |  | | Dominant | Dominant | | Dominant | |  |
| Net monetary benefit* | 27,406 | | 38,174 | 38,627 | | 37,860 | |  |
| ** At cost-effectiveness threshold of one GDP per capita in Vietnam: 2,389 USD per QALY gained ^34^* | | | | | | | | |

1. *Payer perspective*

|  | **PR** | | **SOF/LDV** | **SOF/VEL** | | **SOF+DCV** | |  |
| --- | --- | --- | --- | --- | --- | --- | --- | --- |
| Discounted cost | 4,611 | | 2,317 | 2,101 | | 2,461 | |  |
| Discounted LYs | 17.32 | | 18.17 | 18.19 | | 18.18 | |  |
| Discounted QALYs | 16.20 | | 17.83 | 17.87 | | 17.85 | |  |
| Incremental cost |  | -2,294 | | | -2,509 | | -2,149 | |
| Incremental LYs |  | 0.85 | | | 0.87 | | 0.86 | |
| Incremental QALYs |  | 1.63 | | | 1.66 | | 1.65 | |
| ICER per LY |  | | Dominant | Dominant | | Dominant | |  |
| ICER per QALY |  | | Dominant | Dominant | | Dominant | |  |
| Net monetary benefit* | 34,096 | | 40,287 | 40,581 | | 40,181 | |  |
| ** At cost-effectiveness threshold of one GDP per capita in Vietnam: 2,389 USD per QALY QALY gained ^34^* | | | | | | | | |

**Scenario 2. Discount rates at 1.5% for costs and 1.5% for outcomes**

1. *Societal perspective*

|  | **PR** | | **SOF/LDV** | **SOF/VEL** | | **SOF+DCV** | |  |
| --- | --- | --- | --- | --- | --- | --- | --- | --- |
| Discounted cost | 12,089 | | 4,807 | 4,423 | | 5,155 | |  |
| Discounted LYs | 17.32 | | 18.17 | 18.19 | | 18.18 | |  |
| Discounted QALYs | 16.20 | | 17.83 | 17.87 | | 17.85 | |  |
| Incremental cost |  | -7,282 | | | -7,666 | | -6,934 | |
| Incremental LYs |  | 0.85 | | | 0.87 | | 0.86 | |
| Incremental QALYs |  | 1.63 | | | 1.66 | | 1.65 | |
| ICER per LY |  | | Dominant | Dominant | | Dominant | |  |
| ICER per QALY |  | | Dominant | Dominant | | Dominant | |  |
| Net monetary benefit* | 26,618 | | 37,797 | 38,259 | | 37,487 | |  |
| ** At cost-effectiveness threshold of one GDP per capita in Vietnam: 2,389 USD per QALY gained ^34^* | | | | | | | | |

1. *Payer perspective*

|  | **PR** | | **SOF/LDV** | **SOF/VEL** | | **SOF+DCV** | |  |
| --- | --- | --- | --- | --- | --- | --- | --- | --- |
| Discounted cost | 4,992 | | 2,537 | 2,318 | | 2,680 | |  |
| Discounted LYs | 17.32 | | 18.17 | 18.19 | | 18.18 | |  |
| Discounted QALYs | 16.20 | | 17.83 | 17.87 | | 17.85 | |  |
| Incremental cost |  | -2,455 | | | -2,674 | | -2,312 | |
| Incremental LYs |  | 0.85 | | | 0.87 | | 0.86 | |
| Incremental QALYs |  | 1.63 | | | 1.66 | | 1.65 | |
| ICER per LY |  | | Dominant | Dominant | | Dominant | |  |
| ICER per QALY |  | | Dominant | Dominant | | Dominant | |  |
| Net monetary benefit* | 33,715 | | 40,067 | 40,363 | | 39,962 | |  |
| ** At cost-effectiveness threshold of one GDP per capita in Vietnam: 2,389 USD per QALY QALY gained ^34^* | | | | | | | | |
